# Evaluating the effectiveness of elimination diet in infants with CMP-induced allergic proctocolitis using CoMiSS

**DOI:** 10.1038/s41390-025-04117-7

**Published:** 2025-05-13

**Authors:** Menşure Nur Çelik, Eda Köksal, Sinem Polat Terece, Gizem Köken, Hacer İlbilge Ertoy Karagöl, Arzu Bakirtas

**Affiliations:** 1 Ondokuz Mayis University, Department of Nutrition and Dietetics, Samsun, Türkiye; 2 Gazi University, Department of Nutrition and Dietetics, Ankara, Türkiye; 3 Department of Pediatric Allergy, Gazi University Faculty of Medicine, Ankara, Türkiye

## Abstract

**Background:**

There are no data on using the Cow’s Milk Protein Related Symptom Score (CoMiSS), a clinical screening tool, to monitor symptom improvement in infants with cow’s milk protein allergy (CMPA). This study aimed to evaluate the efficacy of CoMiSS on elimination diet response in infants diagnosed with Cow’s Milk Protein Associated Proctocolitis (CMPAP) compared with healthy controls

**Methods:**

A case-control study with follow-up compared infants with CMPAP on an elimination diet to healthy peers. Infants with CMPAP (*n *= 13) and healthy infants (*n *= 22), aged 17–26 weeks, were included. Four visits were conducted at six, seven, nine, and 12 months of age. CoMiSS was assessed at each visit.

**Results:**

CoMiSS significantly decreased after the four-week elimination diet (*p *< 0.05). At study’s end, total CoMiSS scores increased in both groups (*p *< 0.05). The crying score’s median was higher in CMPAP infants than controls at Visits 1 and 3 (*p *< 0.05). No significant changes were found for other symptoms.

**Conclusions:**

CoMiSS tends to decrease during elimination diet in infants with CMPAP, which is promising for its use in evaluating dietary response. Monitoring the crying score and developing the defecation score specific to CMPAP would serve this purpose more.

**Impact:**

- The CoMiSS can be a promising tool for monitoring responses to elimination diets in infants with proctocolitis, a specific subgroup of CMPA.
- It highlights the potential of CoMiSS to monitor dietary response and suggests refinements for its application, such as focusing on crying scores and developing a CMPA-specific defecation score.
- The findings pave the way for a novel use of CoMiSS beyond its screening purpose, potentially enhancing clinical management of CMPA in infants with proctocolitis. This approach could lead to more tailored and effective dietary interventions, improving patient outcomes and contributing to the refinement of CMPA management tools.

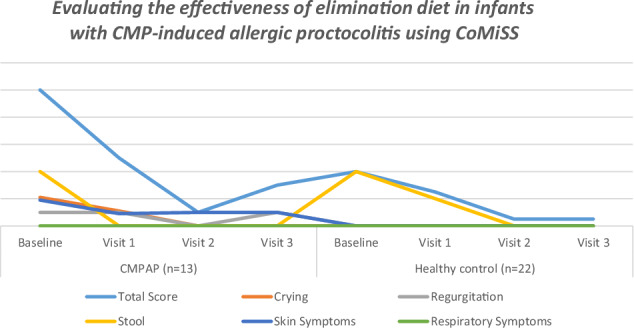

## Introduction

Cow’s milk protein allergy (CMPA) is the most common and most frequently encountered food allergy in the first year of life. It is classified as immunoglobulin (Ig) E-mediated, non-IgE-mediated (Non-IgE) or mixed type.^1^ Non-IgE-mediated CMPA includes food protein-induced enterocolitis syndrome (FPIES), food protein-induced allergic proctocolitis (FPIAP), and food protein-induced enteropathy (FPE).^2,3^

Cow milk is the most common trigger food in FPIAP, characterized by inflammatory changes in the distal colon and rectum triggered by one or more food proteins.^4–7^ The age of first appearance varies between 1 day and six months on average and usually starts between 1 and 4 weeks of life.^8^ Diagnosis requires eliminating the suspect food from the diet, followed by oral provocation.^2^

In recent years, Cow’s Milk-related Symptom Score (CoMiSS) has been developed to be used for screening in cases with suspected CMPA since no biomarker can be used to diagnose CMPA, especially in non-IgE-mediated forms.^9–14^

However, there is no literature study on using CoMiSS to monitor elimination diet response in Cow’s Milk Protein Associated Proctocolitis (CMPAP- This study used the term CMPAP instead of FPIAP since only those diagnosed with cow’s milk protein-associated allergic proctocolitis were evaluated-), which is a specific CMPA subgroup. Therefore, we aimed to evaluate whether CoMiSS could be used to monitor elimination diet response in infants with CMPAP.

## Methods

### Study design and population

In this prospective case-control study, CMPAP infants aged 17–26 weeks who were administered cow’s milk elimination diet and healthy term infants in the same age range who were not administered any elimination diet and were fed age-appropriate nutrition were evaluated. The diagnosis of CMPAP was made in infants aged 0–6 months who were breastfed and/or formula-fed, presented with the complaint of bloody stools and were utterly healthy in other respects, and whose complaint of bloody stools resolved within 48–72 h when cow’s milk and cow’s milk products were removed from the mother’s and/or infant’s diet; and bloody stools recurred within 48–72 h after cow’s milk and cow’s milk products were added back to the mother’s and/or infant’s diet.^15^

The data of the study were collected between August 2020 and March 2022. Approval for this study was obtained from the Gazi University Ethics Commission with the date of 14.07.2020 and meeting number 7. The researcher collected the research data through face-to-face interviews with the mothers of the infants in the sample group.

### Eligibility criteria

Infants aged 17–26 weeks who were breastfed between the study dates and diagnosed with CMPAP by a physician, infants with a gestational age of 37–42 weeks and singleton birth, infants with a birth weight ≥ 2500 g and ≤4500 g, and healthy term infants aged 17–26 weeks who were breastfed were included in the study. The control group consisted of healthy term infants aged 17–26 weeks who were exclusively breastfed, had no history of food allergy or gastrointestinal symptoms, and met the same criteria for gestational age, birth weight, and singleton birth. Premature births, multiple pregnancies, those with a known chronic or systemic disease diagnosed, those with neonatal disease or congenital malformations, those who had an infection or disease requiring hospitalization during the study period, those who never received breast milk, mothers who did not want to continue after participating in the study and their infants were excluded.

### Data gathering

Within the scope of the study, the infants’ general characteristics and health history were questioned with the help of a questionnaire form.

### The cow’s milk protein elimination diet procedure

**Fig. 1:**
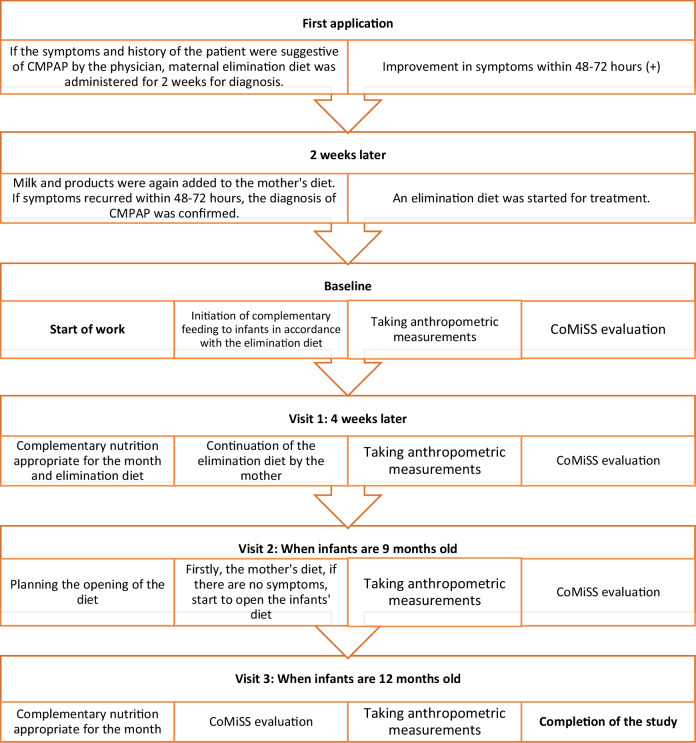
Study protocol.

In the study, infants diagnosed with CMPAP and their mothers remained on an elimination diet under the follow-up of a dietician until the infants were nine months old, and the diet was started to be opened by the milk ladder^16^ from the 9th month. Firstly, the mother’s diet was started, and if it did not cause any symptoms in the infant, the infant’s diet was also started by the milk ladder.^16^ When the infants reached the age of 1 year, dietary elimination was completely terminated. The study protocol is summarized in Fig. 1.

### Evaluation of clinical symptoms: CoMiSS

Clinical symptoms of the infants were evaluated at each visit using CoMiSS. This symptom score evaluates stool pattern, presence and intensity of crying and regurgitation, and skin and respiratory system symptoms. The total score varies between 0 and 33 (Table 1).^9^

**Table 1:** Cow’s milk-related symptom score (CoMiSS)

Symptom	Score	Explanation
Crying (Attacks lasting one week or longer were evaluated)	0 1 2 3 4 5 6	<1 h/day 1–1.5 h/day 1.5–2 h/day 2–3 h/day 3–4 h/day 4–5 h/day >5 h/day
Regurgitation	0 1 2 3 4 5 6	0–2 episodes/day ≥3 to ≤5 of small volüme >5 episodes of >1 coffee spoon >5 episodes of ± half of the feed in <half of the feedings Continuous regurgitations of small volume >30 min after Each feeding Regurgitation of half to complete volume of a feeding in at least half of the feedings Regurgitation of the “complete feeding” after each feeding
Stools (Bristol scale)	4 0 2 4 6	Type 1 and 2 (hard stools) Type 3 and 4 (normal stools) Type 5 (soft stools) Type 6 (liquid stools, if unrelated to infection) Type 7 (watery stools)
Skin symptoms	6 0 0 1 2 3 0 1 2 3	Urticaria (+) No Urticaria (−) No eczema on the head, neck and trunk Mild eczema on the head, neck and trunk Moderate eczema on the head, neck and trunk Severe eczema on the head, neck and trunk No eczema of the extremities Mild eczema of the extremities Moderate eczema of the extremities Severe eczema of the extremities
Respiratory symptoms	0 1 2 3	No respiratory symptoms Slight symptoms Mild symptoms Severe symptoms

### Statistical analysis

The data obtained from the study were analyzed using appropriate statistical methods, using the IBM Statistical Package for the Social Sciences 22.0 (SPSS, version 22.0) statistical package program. The “Mann–Whitney U” test was used when comparing two independent groups since the variables did not show normal distribution. In qualitative data analysis, the “chi-square” test was used to evaluate whether the difference between observed and expected frequencies was significant. All statistical calculations were assessed at a 95% confidence interval and *p *< 0.05 significance level.

## Results

The distribution of the infants, all six months old at the beginning of the study, according to their general characteristics, is given in Table 2. Atopic dermatitis was significantly higher in the CMPAP group than healthy controls (*p *= 0.002). The mean age at onset of CMPAP symptoms was 9.3 ± 6.9 weeks, and the mean age at diagnosis was 12.6 ± 6.1 weeks.

**Table 2:** General information about the infants

	CMPAP (*n *= 13)	Healthy control (*n *= 22)	Total (*n *= 35)	*p*
Sex,female, *n* (%)	4 (30.8)	14 (63.6)	18 (51.4)	0.060
Vaginal birth, *n* (%)	4 (30.8)	7 (31.8)	11 (31.4)	0.948
Birth weight, mean ± SD	3405.8 ± 324.8	3366.6 ± 334.5	3381.1 ± 326.6	0.737
Birth length, mean ± SD	50.4 ± 1.4	50.82 ± 1.7	50.7 ± 1.6	0.445
Atopic dermatitis, *n* (%)	8 (61.5)	2 (9.1)	10 (28.6)	**0.002**

*CMPAP* Cow’s milk protein associated allergic proctocolitis.

Bolded values indicate statistically significant differences (*p* < 0.05).

The median value of the total symptom score was calculated as 10.0 (min–max: 2.0–14.0) in infants with CMPAP and 4.0 (min–max: 0.0–8.0) in healthy controls at baseline (*p *< 0.05). Scores of CoMiSS components and total symptom scores in infants according to months are given in Table 3. At visit 1 (after at least four weeks of elimination diet), the total symptom score of infants with CMPAP decreased compared to the beginning of the study and was found to be higher than healthy controls (*p *< 0.05). There was no difference between the groups regarding total symptom scores at visit 2 (*p *> 0.05). In visit 3, total symptom scores increased in CMPAP and healthy infants compared to the previous visit and were found to be higher in CMPAP infants compared to healthy controls (*p *< 0.05).

**Table 3:** Monthly variation of CoMiSS components and total symptom score in infants

	On elimination diet				Post elimination diet			
	Baseline		Visit 1		Visit 2		Visit 3	
	CMPAP (*n *= 13)	Healthy control (*n *= 22)	CMPAP (*n *= 13)	Healthy control (*n *= 22)	CMPAP (*n *= 13)	Healthy control (*n *= 22)	CMPAP (*n *= 13)	Healthy control (*n *= 22)
	Median (Min–Max)	Median (Min–Max)	Median (Min–Max)	Median (Min–Max)	Median (Min–Max)	Median (Min–Max)	Median (Min–Max)	Median (Min–Max)
Crying	2.0 (0.0–5.0)	0.0 (0.0–2.0)	1.0 (1.0–6.0)	0.0 (0.0–1.0)	0.0 (0.0–3.0)	0.0 (0.0–1.0)	1.0 (0.0–6.0)	0.0 (0.0–1.0)
	*p *= **0.000**		*p *= **0.000**		*p *= 0.578		*p *= **0.038**	
Regurgitation	1.0 (0.0–6.0)	0.0 (0.0–1.0)	1.0 (0.0–5.0)	0.0 (0.0–4.0)	0.0 (0.0–0.0)	0.0 (0.0–1.0)	1.0 (1.0–6.0)	0.0 (0.0–1.0)
	*p *= 0.067		*p *= 0.085		*p *= 0.827		*p *= 0.489	
Stools	4.0 (0.0–6.0)	4.0 (0.0–6.0)	0.0 (0.0–4.0)	2.0 (0.0–4.0)	0.0 (0.0–4.0)	0.0 (0.0–4.0)	0.0 (0.0–4.0)	0.0 (0.0–4.0)
	*p *= 0.180		*p *= 0.489		*p *= 0.749		*p *= 0.960	
Skin Symptoms	2.0 (1.0–2.0)	0.0 (0.0–6.0)	1.0 (0.0–2.0)	0.0 (0.0–2.0)	0.0 (0.0–2.0)	0.0 (0.0–1.0)	0.0 (0.0–6.0)	0.0 (0.0–1.0)
	*p *= **0.000**		*p *= 0.053		*p *= 0.149		*p *= 0.139	
Respiratory symptoms	0.0 (0.0–0.0)	0.0 (0.0–0.0)	0.0 (0.0–0.0)	0.0 (0.0–1.0)	0.0 (0.0–1.0)	0.0 (0.0–1.0)	0.0 (0.0–1.0)	0.0 (0.0–1.0)
	*p *= 1.000		*p *= 0.674		*p *= 0.880		*p *= 0.827	
Total score	10.0 (2.0–14.0)	4.0 (0.0–8.0)	5.0 (1.0–11.0)	2.5 (0.0–9.0)	1.0 (0.0–7.0)	0.5 (0.0–5.0)	3.0 (0.0–14.0)	0.5 (0.0–8.0)
	*p *= **0.001**		*p *= **0.045**		*p *= 0.533		*p *= **0.049**	

*CMPAP* Cow’s milk protein associated allergic proctocolitis.

Bolded values indicate statistically significant differences (*p* < 0.05).

At the beginning of the study and Visit 1, the median crying score was significantly higher in infants with CMPAP than healthy controls (*p *< 0.05). In Visit 2, the crying score median value tended to decrease, and the difference between infants with CMPAP and those in healthy controls was not significant (*p *> 0.05). In visit 3, the crying score was significantly higher in infants with CMPAP than healthy controls (*p *< 0.05). While no significant change was determined in terms of other symptoms during the study, only skin scores were higher than healthy controls at the beginning of the study (*p *< 0.05).

**Fig. 2:**
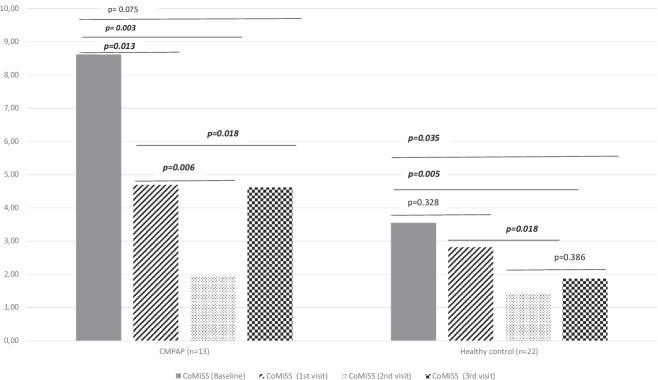
Variation of CoMiSS symptom scores within the group.

Figure 2 shows the changes in CoMiSS scores at four visits in both CMPAP infants and healthy controls. In the CMPAP group, CoMiSS scores decreased significantly from baseline to visit 1 (*p *= 0.013) and from baseline to visit 2 (*p *= 0.03). Although the decrease from baseline to visit 3 was not statistically significant (*p *= 0.075), a significant decreasing trend was observed. Comparisons between successive visits showed significant decreases between visits 1 and 2 (*p *= 0.006), followed by an increase from visit 2 to visit 3 (*p *= 0.018). In the healthy control group, CoMiSS scores also decreased significantly from baseline to visit 2 (*p *= 0.005) and from baseline to visit 3 (*p *= 0.035).

The change in CoMiSS during the study period is given in Table 4. Accordingly, there was a decrease of −3.92 ± 4.31 (27.33%) in the CoMiSS scores of infants with CMPAP between baseline and visit one and a decrease of −6.69 ± 4.39 points (68.08%) between baseline and visit 2. A significant difference was found between the groups regarding the decreases between baseline and visit one and between baseline and visit 2 (*p *< 0.05).

**Table 4:** Change in CoMiSS during the study

CoMiSS change	CMPAP (*n *= 13)	Healthy control (*n *= 22)	*p*
	x̄ ± SD	x̄± SD	
Visit 1-Baseline	−3.92 ± 4.31	−0.73 ± 3.68	0.031*
Visit 2-Baseline	−6.69 ± 4.39	−2.14 ± 2.92	0.003*
Visit 3-Baseline	−4.00 ± 6.88	−1.68 ± 3.36	0.106

*CMPAP* Cow’s milk protein associated allergic proctocolitis.

Values marked with * indicate statistically significant differences (*p* < 0.05).

## Discussion

It has been shown in many studies that CoMiSS can be used for screening when CMPA was previously suspected.^17–19^ However, CoMiSS was evaluated for the first time to monitor response to treatment in a specific subgroup of CMPAP diagnosed in a pediatric allergy clinic. As known, CMPAP is the most common form of CMPAP, and in our study, it was shown for the first time that CoMiSS can be used to monitor response to diet in non-IgE-mediated CMPAP.

A comparison of CoMiSS scores with healthy controls was used in only one other study apart from ours.^13^ In a recent study, only healthy 6–12-month-old infants from six European countries were analyzed for prospective CoMiSS evaluation; CoMiSS median values were determined as 3 (1–5). The median values of the neighborhood scores of the infants according to months were reported as 4 in 6-month-old infants, 3 in 7- and 9-month-old infants, and 1 in 12-month-old infants.^20^ These results were similar in healthy infants evaluated in our study (Table 3).

The difference in the total symptom score between healthy and CMPAP infants during our study was mainly due to the crying score (Table 3). It was observed that the crying score in the CoMISS tended to decrease from baseline in infants with CMPAP compared to healthy infants. The persistence of a significant difference in the crying score at Visit 1 (first control after the elimination diet) and Visit 3 (after the elimination diet was switched on) compared to healthy controls suggests that inflammation at the tissue level in the gastrointestinal tract has not yet been controlled in infants with CMPAP, despite the absence of clinically bloody, mucusy stool. The infant is eventually directly exposed to milk protein after heat-treated and fermented products by the milk ladder, even if the diet is switched on.

In our study, we focused on the inflammation hypothesis regarding the persistence of crying symptoms in infants with CMPAP. However, the pathogenesis of colic is multifactorial and may include dysbiosis, intestinal motility disorders, and other mechanisms.^21^ Therefore, it is thought that the relationship between crying symptoms and these different mechanisms should be evaluated more comprehensively.

Although we did not use fecal calprotectin or a similar parameter to support this, we think that crying may indirectly indicate inflammation in the gastrointestinal tract in CMPAP. Thus, although these infants with CMPAP did not produce bloody or mucous stool when their elimination diet was stopped at one year of age to check the development of tolerance, they again showed a significant increase in crying scores compared to healthy controls, indicating that they may still not have fully developed tolerance to cow’s milk. Another explanation for the rise in crying score only in infants with CMPAP compared to healthy controls after the elimination diet was switched on may be that although the mother’s diet was switched on first. Then the infant’s diet was switched on, there was eventually direct exposure to milk protein after heat-treated and fermented products by the milk ladder.

In fact, we expected a difference in the defecation score in CoMiSS compared to healthy controls both at the beginning of elimination diet treatment in CMPAP and in the follow-up. We think that the similarity of the defecation score to healthy controls in patients with CMPAP is related to the evaluation of only stool consistency (hard, soft, etc.) in this scoring. The major clinical finding in CMPAP is frequent bloody mucous stools. However, in the current CoMiSS, the frequency of defecation and bloody/mucous stools are not evaluated in the defecation score. Therefore, we think that CoMiSS should be improved in this respect. However, we would not expect the other components of the CoMiSS, such as regurgitation, skin, and respiration, to be different in infants with CMPAP than in healthy controls without concomitant eosinophilic oesophagitis, atopic dermatitis, IgE-mediated food allergy or asthma. Moreover, approximately two-thirds of our CMPAP cases were accompanied by atopic dermatitis, which explains the higher skin symptom score on the CoMiSS at baseline compared to healthy control.

Previously, Köse et al. compared the CoMiSS scores before and after the elimination diet in 112 infants diagnosed with milk and egg allergy classified as IgE-mediated, non-IgE-mediated, and mixed-type food allergy. In the same study, although there were significant decreases in CoMiSS scores after the diet compared to baseline in all three food allergy groups, no difference was found between the groups regarding this change.^22^ According to a recent meta-analysis, CoMiSS may be a promising symptom score in diagnosing CMPA and a useful tool for monitoring response to an elimination diet.^23^ However, no consensus has yet been reached on the cut-off for diagnosis and treatment.^24^ In 2021, the developers of the CoMiSS scale emphasized that the CoMiSS scale is guiding in the diagnosis phase by showing a decrease of more than 50% in the mean CoMiSS score after three weeks of diet therapy compared to baseline in infants with suspected CMPA in primary care.^17^ When the decreases in CoMiSS scores were analyzed in our study, the reductions after four weeks (visit 1) and 12 weeks (visit 2) of diet treatment compared to baseline were higher than the decreases in healthy infants (*p *< 0.05) (Table 4). Similar to the study of Vandenplas et al.^17^ in infants with CMPA, the fact that CoMiSS scores decreased by more than 50% during the elimination diet (visit 1 and visit 2) in this study suggests that this symptom score may be necessary in evaluating the effectiveness of diet therapy. The significant change in CoMiSS scores between baseline and visit 2 probably reflects the combined effects of the prolonged elimination diet and the re-entry phase, which may have elicited different symptom responses. This observation emphasises the dynamic nature of symptom improvement and the importance of monitoring dietary interventions over an extended period.

In clinical practice, CoMiSS can be a useful tool for monitoring dietary response, especially when tracking symptoms over time. While there are no universally established thresholds for CoMiSS scores, we believe that significant changes in symptom scores, particularly those indicating a reduction in crying or defecation abnormalities, could guide clinicians in adjusting the diet. CoMiSS can also be helpful for long-term management of CMPA on and response to diet. Clinicians can use it to determine if symptoms are improving or if further dietary changes are needed, such as reintroducing milk proteins or modifying other aspects of the diet. Because understanding the changing nutritional and immunological needs of these infants is essential for effective CMPA management, tools like CoMiSS can offer valuable insights into this ongoing process. Therefore, while CoMiSS is valuable for short-term monitoring, further studies with longer follow-up periods are needed to assess its long-term applicability in CMPAP management.

The strengths of our study are the study of CoMISS in a specific CMPA subgroup, comparison with healthy controls, and evaluation of whether the primary study parameter CoMISS can be used in the follow-up of response to treatment. Our study’s most important limiting aspect is that a dietitian saw patients with CMPAP, and CoMISS was applied at least two weeks after starting the elimination diet due to the pandemic. Therefore, the CoMiSS defecation score in the CMPAP group may not have been different from healthy subjects. Another limitation may be the sample size of the CMPAP case group, but this was generally associated with the decrease in the number of patients cared for during the pandemic. The data collection period coincided with the COVID-19 pandemic, which led to hesitancy among patients to visit hospital settings. Additionally, the long follow-up duration and the transition to complementary feeding caused some mothers to be reluctant to participate in the study, or they withdrew as the study progressed.

In conclusion, our study supports using CoMiSS to monitor elimination diet response in infants with CMPAP. It is a useful tool for monitoring dietary responses in infants with CMPAP, especially during the early stages of an elimination diet. Monitoring the crying score and developing a CMPAP-specific defecation score would serve this purpose better. However, the increase in scores observed at the end of the study highlights the need for further research to determine its long-term reliability and clinical relevance. Future studies should investigate whether CoMiSS can reliably guide dietary adjustments and symptom monitoring beyond the initial treatment phase.

## Data Availability

The datasets generated during and/or analyzed during the current study are available from the corresponding author on reasonable request.
